# High Fixation Failure Rate of Cephalomedullary Nail Fixation in Patients with Low-Energy Basicervical Femoral Fractures: Do We Need Extramedullary Reduction?

**DOI:** 10.3390/medicina61010112

**Published:** 2025-01-14

**Authors:** Chang-Jin Yon, Ki-Cheor Bae, Young-Hun Kim, Kyung-Jae Lee

**Affiliations:** Department of Orthopedic Surgery, Keimyung University Dongsan Hospital, Keimyung University School of Medicine, Daegu 42601, Republic of Korea; poweryon@dsmc.or.kr (C.-J.Y.); bkc@dsmc.or.kr (K.-C.B.); kyhkim4190@naver.com (Y.-H.K.)

**Keywords:** basicervical femoral fracture, fixation failure, cephalomedullary nail, extramedullary reduction

## Abstract

*Background and Objectives:* A basicervical femoral fracture is a relatively uncommon type of proximal femoral fracture. However, as the proportion of proximal femoral fractures rises in conjunction with the aging of society, the absolute number of patients with basicervical femoral fractures is also increasing. Nevertheless, the optimal surgical methods for the treatment of basicervical femoral fractures remain a topic of debate. The aim of this study is to evaluate the failure rates of cephalomedullary nail fixation in basicervical femoral fractures based on reduction types. *Methods:* A retrospective analysis was conducted on 22 patients (22 hips) with AO/OTA 31-A1.2 hip fractures who had undergone treatment with a cephalomedullary nail (PFNA-II or Gamma-3) between March 2007 and February 2018. They were classified into three groups based on the reduction types: extramedullary (E), anatomical (A), or intramedullary (I). The intramedullary group included cases where the basicervical component was impacted into the medullary canal, while the extramedullary group comprised cases where the component was displaced beyond the medullary canal. The anatomical group consisted of specimens that exhibited complete anatomical reduction. This was determined by both the anteroposterior (AP) view and the lateral view using simple radiographs. *Results:* There were 13 patients (59.1%) in Group E and 9 patients (40.9%) in Group A. No patients were classified in Group I. Fixation failure occurred in four patients (18.1%, 4/22). In Group E, no patients exhibited fixation failure (0%, 0/13). In contrast, four patients in Group A demonstrated fixation failure (44.4%, 4/9). Group A exhibited a significantly higher incidence of fixation failure (0% vs. 44.4%, *p* =0.037) compared to Group E. *Conclusion:* In the treatment of low-energy basicervical femoral fractures with cephalomedullary nails, extramedullary reduction demonstrated a lower rate of fixation failure compared to anatomical reduction in this study. While definitive conclusions regarding its superiority cannot be drawn due to the limited sample size, extramedullary reduction may serve as a promising alternative to reducing the high fixation failure rate associated with this challenging fracture type.

## 1. Introduction

Basicervical femoral fractures constitute a relatively rare subset of proximal femoral fractures, comprising 1.8% to 7.6% of cases reported in the literature [1,2]. There is no consensus in the literature regarding the description of this type of fracture. For instance, the term ’basicervical’ is frequently used in conjunction with terms like ’femur intertrochanteric fracture’, ’neck fracture’, and ’pertrochanteric fracture’ to describe this type of fracture [3,4,5,6,7,8]. The variation in terminology likely stems from the anatomical positioning of the basicervical region. There is no consensus on the definition of a basicervical fracture. The most commonly referenced definition describes it as a proximal femoral fracture occurring at the base of the femoral neck, where it joins the intertrochanteric area [3,4]. A recent study refined the localization of the fracture line to the base of the femoral neck, medial to the intertrochanteric line, extending above the lesser trochanter but positioned more laterally than a typical transcervical fracture [9].

There is still no consensus on the optimal treatment approach for basicervical femoral fractures. As indicated in published reports, the success rate for the treatment of basicervical femoral fractures with a sliding hip screw was found to be relatively low. In contrast, certain studies have documented positive outcomes with cephalomedullary nail fixation [10,11]. At the present time, cephalomedullary nail fixation is the most preferred method for the treatment of pertrochanteric femoral fractures [12]. Nevertheless, Watson et al. [13] documented a notable failure rate in managing basicervical femoral fractures using cephalomedullary nails, thereby demonstrating that the choice of cephalomedullary nail remains a point of debate in the context of basicervical femoral fracture management. A substantial body of research has concentrated on the surgical outcomes associated with implant selection. It is challenging to identify studies that examine the impact of objective reduction states on the outcomes of basicervical fractures.

The purpose of this study is to analyze the failure rate in basicervical femoral fractures managed with cephalomedullary nail fixation, according to the reduction types.

## 2. Materials and Methods

### 2.1. Patient Data Collection

Ethical approval for this retrospective study was obtained from the institutional review board (No. 2020-03-039). A retrospective radiographic review was conducted of all patients who underwent osteosynthesis for proximal femoral fractures at our institution between March 2007 and February 2018. A three-dimensional computed tomography (3D CT) scan was conducted on all patients presenting with a proximal femoral fracture prior to surgical intervention.

**Figure 1:**
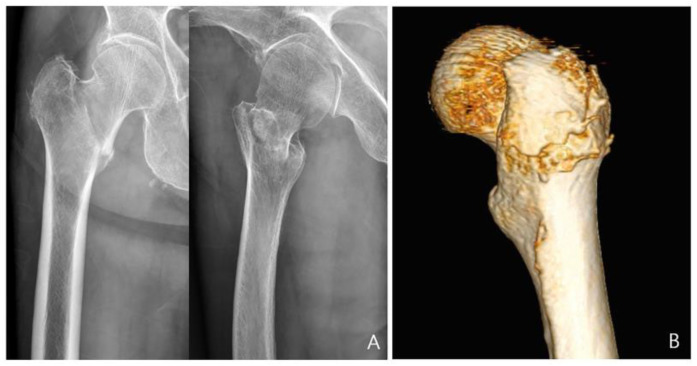
Radiographic images of a patient with a basicervical femoral fracture. In the anteroposterior view, the fracture appears as a two-part fracture, and the comminution of the greater trochanter is not distinctly visible in the lateral view (**A**). A minimally displaced fracture of the greater trochanter is evident on the 3D CT image (**B**).

This study focused on patients with a basicervical femoral fracture, defined as a two-part fracture at the base of the femoral neck that was medial to the intertrochanteric line and exited above the lesser trochanter, but more lateral than a classic transcervical fracture. In comparison to plain radiographs, computed tomography (CT) images demonstrated the presence of minor fractures in the greater trochanter in a subset of patients who had been initially diagnosed with pure two-part femoral neck fractures based on plain radiographs (Figure 1). Taking these factors into account, this study included not only patients with simple two-part basicervical femoral fractures but also those with minimally displaced small fragments of the greater trochanter identified on CT scans.

All patients included in this study met the following criteria: (1) aged 60 years or older; (2) living independently at home prior to the injury; (3) basicervical femoral fractures (AO/OTA 31-A1.2) resulting from a low-energy mechanism (non-pathologic origin); (4) no history of mental health disorders or cognitive impairments; (5) treated with cephalomedullary nails; and (6) a minimum follow-up period of 12 months postoperatively. Patients were excluded if they met any of the following criteria to maintain the focus on basicervical femoral fractures treated with cephalomedullary nails: (1) fractures caused by high-energy trauma or pathologic fractures; (2) history of previous surgical intervention on the affected hip prior to the index operation; (3) treatment with implants other than cephalomedullary nails, such as dynamic hip screws or arthroplasty; and (4) follow-up duration of less than 12 months, resulting in insufficient data for analysis.

**Figure 2:**
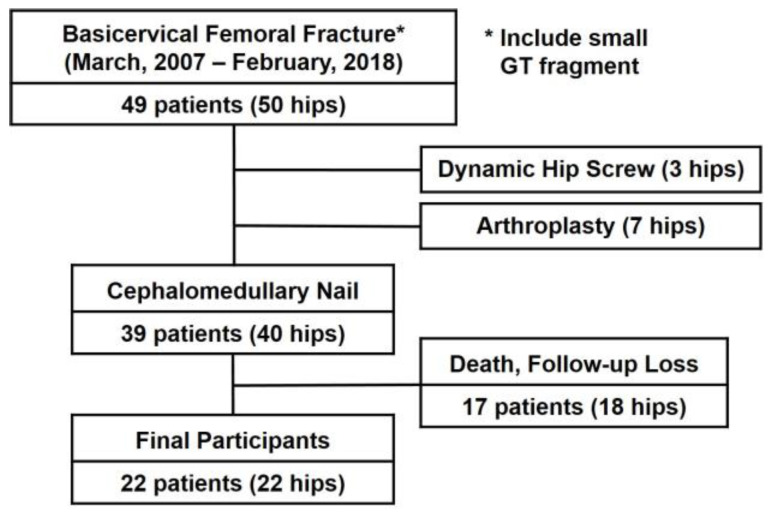
Patient selection process.

Consequently, a total of 49 patients (50 hips) were included in the radiographic review. This study excluded three cases treated with a dynamic hip screw and seven cases managed with hip arthroplasty for basicervical femoral fractures. Osteosynthesis with a cephalomedullary nail was performed in 39 patients (40 hips) with basicervical femoral fractures. Of these, 17 patients (18 hips) either passed away or were lost to follow-up during the study period. Finally, 22 patients (22 hips) who had undergone treatment with a cephalomedullary nail (PFNA-II or Gamma-3) for a basicervical femoral fracture and had been followed up for a minimum of one year were selected for analysis of postoperative outcomes (Figure 2).

### 2.2. Surgical Procedures

All surgical procedures were conducted with patients positioned supine on a fracture table under general or spinal anesthesia. A single senior surgeon with over 15 years of experience in hip surgery conducted all the operations. Standard closed reduction techniques, including abduction, traction, and internal rotation, were utilized to align the fracture, with alignment confirmed via fluoroscopy. In the situation that closed reduction was deemed unsatisfactory, particularly when the proximal (head and neck) fragment was positioned intramedullary (on the lateral side of the medial cortex of the distal fragment) in the anteroposterior (AP) view and there was evidence of posterior sagging or posterior neck displacement in the cross-table lateral view, intraoperative manipulation was conducted later through the entry incision using a Hohmann retractor or curved Kelly forceps. The placement of lag screws or helical blades was conducted in accordance with the recommendations of Baumgartner et al., which stipulate a tip apex distance (TAD) of less than 25 mm to mitigate the risk of cut-out. As defined by Baumgaertner et al., the tip apex distance (TAD) is calculated as the sum of the distances, in millimeters, from the tip of the lag screw to the apex of the femoral head as measured on both anteroposterior and lateral radiographs, with corrections applied for magnification [14]. The screws were positioned in a center–center or inferior–center configuration [15].

### 2.3. Radiological Classification 

The pelvis AP and cross-table lateral radiographs were taken after surgery. Standard AP radiographs of the hip were obtained with both legs positioned to an internal rotation of 15°. The postoperative cross-table lateral radiographs were taken with the contralateral hip flexed and abducted.

**Figure 3:**
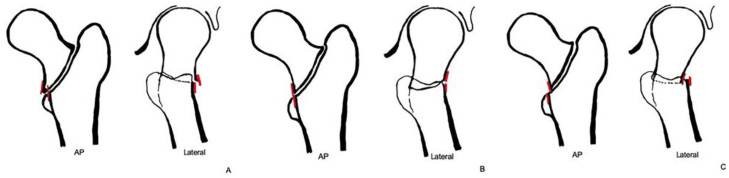
Diagram illustrating the classification of reduction states based on AP and lateral views. (**A**) Extramedullary reduction. (**B**) Anatomical reduction. (**C**) Intramedullary reduction.

All participants were classified into three groups based on the reduction types: extramedullary (E), anatomical (A), or intramedullary (I) (Figure 3).

The extramedullary group included cases where the basicervical component was displaced beyond the medullary canal, in an AP view, and where the basicervical component was noted to be situated on the anterior side of the anterior cortex of the distal fragment, in a lateral view. In the AP view, the criterion was that the inner line of the proximal fragment cortex was situated more medially than the outer line of the distal fragment cortex. In the lateral view, the criterion was that the inner line of the proximal fragment cortex was situated more anteriorly than the outer line of the distal fragment on the anterior side of the femur.

The intramedullary group comprised cases where the basicervical component was observed to be impacted into the medullary canal in an anteroposterior (AP) view, and where the basicervical component was noted to be situated on the posterior side of the anterior cortex of the distal fragment in a cross-table lateral view. In the AP view, the criterion was that the outer line of the proximal fragment cortex was situated more laterally than the inner line of the distal fragment cortex. In the lateral view, the criterion was that the outer line of the proximal fragment cortex was situated more posteriorly than the inner line of the distal fragment on the anterior side of the femur.

The anatomical group consisted of specimens that exhibited complete anatomical reduction. In the AP view, the criterion was that the medial cortex of the proximal and distal fragments should overlap without deviating by more than one cortex. In the lateral view, the criterion was that the anterior cortex of the proximal and distal fragments should overlap without deviating by more than one cortex.

### 2.4. Failure of Fixation 

**Figure 4:**
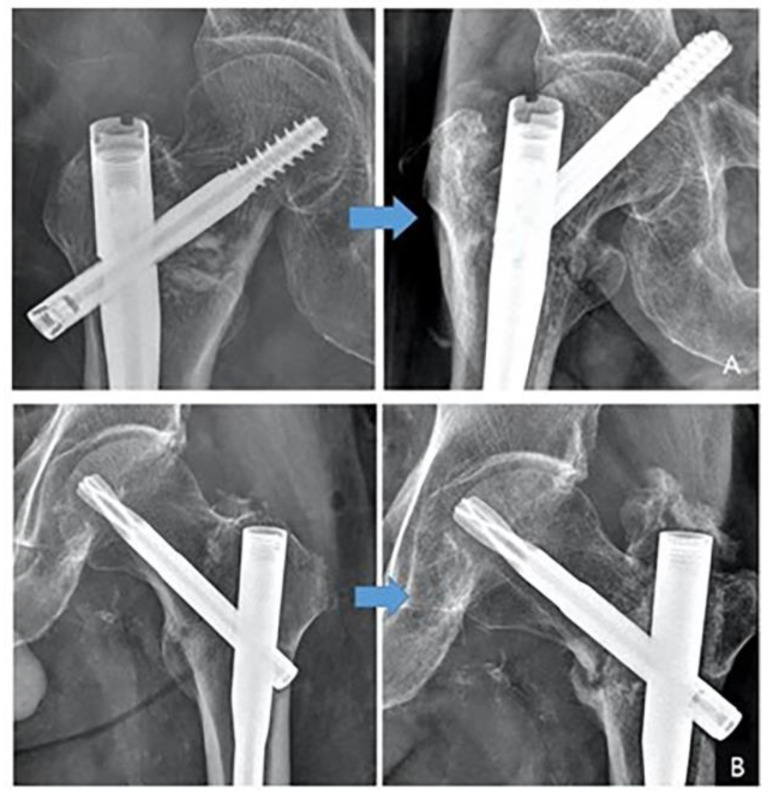
Two types of fixation failure identified in basicervical fractures. (**A**) Cut-through or cut-out of the lag screw. (**B**) Rotational instability leading to nonunion.

Failure of fixation was identified in two distinct modes (Figure 4). One type of mode of failure is substantial collapse, accompanied by a lag screw or helical blade sliding. Ultimately, contact between the subcapital region and the nail results in screw cut-out or cut-through.

The other type of mode of failure is nonunion of the fracture (no sign of healing radiographically or clinically at 6 months postoperatively). This phenomenon may be attributed to the nail’s role as an intramedullary buttress, which serves to prevent collapse. In the long term, this results in rotational and angular failure, which in turn causes the nonunion to occur. Radiographs of patients included in the study series were reviewed by two orthopedic surgeons. Revision operation was performed for fixation failures after the index operation.

### 2.5. Statistical Analysis

All statistical analysis was conducted using IBM SPSS version 23.0 (IBM Co., New York, NY, USA). Statistical analyses between the two study groups were conducted using the Mann–Whitney U test, Chi-squared test, or Fisher’s exact test. A *p*-value below 0.05 was deemed statistically significant.

## 3. Results

A total of 22 patients (22 hips) were included in this study of basicervical femoral fractures. In the AP view, 13 patients (59.1%) were categorized into Group E and nine patients (40.9%) into Group A. The mean extramedullary displacement in Group E, defined as the distance from the inner cortex of the proximal fragment to the outer cortex of the distal fragment, was 1.37 mm (range, 0.12–3.38 mm). No patients were classified in Group I. In the lateral view, all patients demonstrated an identical reduction status, classified as anatomical reduction (Group A). Ultimately, the AP view allowed for the division of the 22 patients into two distinct groups. The first group, comprising 13 patients, exhibited extramedullary reduction characteristics and was designated as Group E. The second group, comprising nine patients, demonstrated anatomical reduction characteristics and was designated as Group A. There were no statistically significant differences in age, body mass index (BMI), or T-score on the contralateral femoral neck in the Bone Mineral Density (BMD) test or follow-up period between the two groups (Table 1).

Four patients (18.1%, 4/22) experienced fixation failure. No patients in Group E experienced fixation failure, yielding a 0% failure rate (0/13). In Group A, the fixation failure rate was 44.4% (4/9), showing a statistically significant difference compared to Group E (p = 0.037). Among these cases, the modes of failure included cut-through (two cases) and nonunion caused by rotational instability (two cases) (Table 2).

Two additional cases involved complications unrelated to fixation failure. In the anatomical group, one patient experienced a distal femoral fracture treated with plate osteosynthesis. In the extramedullary group, one case of avascular necrosis of the femoral head required conversion to arthroplasty.

## 4. Discussion

Basicervical femoral neck fractures are known to have a high rate of fixation failure following internal fixation surgery. In this study, we observed that fixation failure remained relatively frequent in basicervical femoral fractures treated with cephalomedullary nails (CMNs), even when the tip apex distance (TAD) and alignment were within acceptable ranges. This finding suggests that factors beyond implant positioning may play a critical role in the surgical outcomes of these fractures, specifically the reduction technique used.

Previous studies have raised concerns regarding the appropriateness of CMNs in basicervical fractures [16]. Watson et al. reported a high rate of fixation failure in two-part basicervical femoral fractures treated with CMNs, despite achieving anatomical or near-anatomical reduction in all cases [13]. Similar results were observed in our study, where the failure rate was also elevated, even when near-anatomical reductions were achieved. While the criteria for near-anatomical reduction were not clearly defined in Watson’s study, our findings suggest that achieving anatomical reduction alone may not be sufficient to prevent fixation failure in this fracture type.

Hu et al. demonstrated favorable outcomes with CMN use in basicervical fractures, but their study involved a younger cohort with a more diverse range of fracture patterns [17]. This raises the possibility that patient age and fracture complexity may significantly influence outcomes. In contrast, our cohort included older patients, and all fractures were classified as two-part basicervical fractures, which may account for the higher failure rate observed.

The biomechanical instability of basicervical fractures may also explain the higher incidence of collapse observed in these fractures compared to intertrochanteric fractures [8,18]. Basicervical fractures are located closer to the femoral neck, a region that bears higher loads and is inherently less stable, potentially leading to greater risk of fixation failure. This is consistent with findings by Ryu et al., who identified intramedullary reduction as a risk factor for fixation failure in intertrochanteric fractures treated with CMNs [19]. In light of this, we made concerted efforts to avoid intramedullary reduction and instead aimed for extramedullary reduction in our cohort. Recent research has shown that the Wayne County reduction technique outperforms anatomical reduction in managing unstable comminuted intertrochanteric femoral fractures [20,21]. The principle of Wayne-County reduction technique involves displacing the distal fracture fragment outward to align or overlap the medial cortical bone, while engaging the posterior cortical bone to form a posteromedial buttress. This configuration enhances fracture stability and helps prevent pronation and supination deformities. Additionally, the valgus alignment reduces the lever arm forces acting on the fracture, thereby minimizing bending stresses [21]. The radiographic characteristics of the Wayne County reduction closely resemble those of basicervical femoral fractures categorized as the extramedullary reduction group in this study. Considering the greater biomechanical instability of basicervical fractures compared to intertrochanteric fractures, it is essential to acknowledge and address their high risk of fixation failure [8,22,23].

In our analysis, extramedullary reduction appeared to stabilize the proximal bone fragment via the anteromedial calcar region, potentially reducing the risk of varus collapse and fixation failure. This supports the hypothesis that extramedullary reduction, which closely mirrors the principles of the Wayne County technique, may offer better biomechanical stability in basicervical fractures, where intramedullary techniques often lead to higher failure rates.

The choice of implant also appears to influence outcomes in basicervical femoral fractures. Kim et al. reported improved outcomes using blade-type or dual integrated screw-type lag screws compared to single screw-type lag screws in CMN-treated basicervical fractures [24]. Sharma et al. further demonstrated that proximal femoral nails (PFNs) offered greater stability than cancellous cannulated screws (CCSs), although they were associated with a higher rate of technical errors [25]. In biomechanical testing, Kwak et al. found that while helical blades were prone to rotational instability and varus collapse, screw blade hybrid systems offered better resistance to proximal fragment migration [26].

Our findings align with those of Xiong et al., who suggested that a sliding hip blade with a side plate is the optimal fixation method for basicervical fractures [27]. It has been demonstrated that the application of a Wayne County reduction technique is more effective than the use of an anatomical reduction technique in the treatment of unstable intertrochanteric femoral fractures with comminution. The morphology of the Wayne County reduction on plain radiographs resembles that of basicervical femoral fractures categorized as the extramedullary reduction group in this study. Given the increased instability of basicervical femoral fractures in comparison to intertrochanteric femoral fractures, it is essential to recognize and consider the high probability of fixation failure. Extramedullary reduction is hypothesized to stabilize the proximal bone fragment through the anteromedial calcar region, potentially lowering the risk of fixation failure.

This study included pure two-part basicervical fractures as well as cases with small, minimally displaced fragments of the greater trochanter. This inclusion may complicate direct comparisons with other studies. However, 3D CT scans were conducted for all patients to analyze preoperative fracture patterns, revealing minimal displacement of the greater trochanter in cases initially identified as simple two-part fractures on plain radiographs. Based on these findings, such cases were incorporated into this study. Additionally, this study introduces a classification method for objectively evaluating postoperative reduction states, which sets it apart from previous research in this area.

The limitations of this study include its relatively small patient cohort and retrospective design, which inherently restrict the ability to draw generalizable conclusions. Additionally, two distinct implant types (PFNA-II and Gamma-3) were utilized rather than a single implant type, and the number of cases was insufficient for a comprehensive analysis of the differences in surgical outcomes between these implants.

Furthermore, several factors that may influence fixation failure, such as alignment, implant position, bone quality, and implant design, were not fully standardized due to the retrospective nature of this study and the rarity of basicervical femoral fractures. While we attempted to address bone quality by including preoperative BMD measurements, this study’s design limited our ability to systematically evaluate and control for these variables.

Future prospective studies with larger patient cohorts are necessary to overcome these limitations and to provide more robust and generalizable findings.

## 5. Conclusions

In this study, extramedullary (E) reduction was associated with a lower fixation failure rate compared to anatomical (A) reduction in the treatment of basicervical femoral fractures using cephalomedullary nails. While the findings suggest that extramedullary reduction may enhance biomechanical stability and reduce the risk of fixation failure, the limited sample size precludes definitive conclusions regarding its superiority over anatomical reduction.

However, these results highlight the potential of extramedullary reduction as an alternative method to addressing the high fixation failure rate observed in basicervical femoral fractures. Further studies with larger cohorts are necessary to validate these findings and to establish optimal surgical strategies for managing this challenging fracture type.

## Figures and Tables

**Table 1:** Demographic data.

	Group E	Group A
Number of patients	13	9
Gender (Male: Female)	12: 1	4: 5
Fixation implant (PFNA II: Gamma-3)	11: 2	6: 3
Age (years)	76.6 (70–85)	76.4 (52–83)
Body mass index (kg/m^2^)	21.0 (7.1–21.9)	23.1 (7.1–20.1)
T-score on contralateral femoral neck in BMD	−2.9 (−4.1–−2.5)	−2.8 (−3.9–−2.2)
Length of follow-up (months)	28.7 (12–64)	30.6 (12–46)

Group E: Extramedullary reduction group, Group A: Anatomical reduction group, BMD: Bone Mineral Density.

**Table 2:** Patient data on fixation failures.

Case	Age (Year)	Sex	Study Group (Reduction)	Implant	Tip Apex Distance (mm)	Mode of Failure	Intervention
1	89	F	A, Anatomical	Gamma-3	19.2	Cut-through	Hemiarthroplasty
2	86	F	A, Anatomical	PFNA-II	12.1	Nonunion	No intervention due to lower function
3	77	F	A, Anatomical	Gamma-3	19.4	Cut-through	Total hip arthroplasty
4	66	M	A, Anatomical	PFNA-II	10.9	Nonunion	Total hip arthroplasty

## Data Availability

The datasets generated during the current study are not publicly available due to the patient privacy law but are available from the corresponding author on reasonable request.
